# Rectal Injury Due to the Incorrect Insertion of a Vagi-Pipe Into the Rectum During Total Laparoscopic Hysterectomy: A Case Report

**DOI:** 10.7759/cureus.78219

**Published:** 2025-01-29

**Authors:** Takashi Natsume, Mayumi Kobayashi-Kato, Yasuhito Tanase, Masaya Uno, Mitsuya Ishikawa

**Affiliations:** 1 Department of Gynecology, National Cancer Center Hospital, Tokyo, JPN

**Keywords:** incorrect insertion, laparoscopic low anterior resection, rectal injury, total laparoscopic hysterectomy, vagi-pipe

## Abstract

Total laparoscopic hysterectomy (TLH) is the most common gynecologic surgery. The use of a vaginal pipe to facilitate TLH is a popular method for enabling incision into the vaginal canal. However, this device is associated with serious complications. We report the case of a patient who underwent laparoscopic low anterior resection due to rectal injury caused by the incorrect insertion of a Vagi-Pipe (Hakko Co. Ltd., Nagano, Japan) into the rectum during TLH for endocervical positive margins after conization of cervical intraepithelial neoplasia. In the present case, the narrow vaginal orifice made it difficult for the assistant surgeon to distinguish between the vagina and anus. Based on this experience, a change was made at our institution to cover the anus with a cloth to prevent misidentification. Surgeons performing TLH should be mindful that the use of a vaginal pipe can lead to incorrect insertion into the rectum.

## Introduction

Total laparoscopic hysterectomy (TLH) is one of the most frequently performed gynecologic surgeries. It has been reported that the number of TLHs has increased every year, and in 2016, 16,940 cases of TLH were performed in Japan [1]. Although TLH has the advantages of smaller scars, less bleeding, less postoperative pain, a shorter hospital stay, and early postoperative recovery compared with open hysterectomy, intraoperative complications are reported to occur more frequently with laparoscopy [2]. Complications of hysterectomy can involve the bladder, ureter, and rectum [3-5]. The incidence of ureteral injury ranges from 0.21% to 2% during laparoscopic hysterectomy and approximately 0.04% during all gynecologic open surgery [6-9]. The incidence of rectal injuries has been reported to be 1.5% in laparoscopic surgery, compared to 0.3% in all open surgery in gynecology [10,11]. However, to date, there have been few cases of rectal injury during TLH in the absence of rectal invasion by cancer or endometriosis [12]. We present a case of rectal injury due to the incorrect insertion of a Vagi-Pipe (Hakko Co. Ltd., Nagano, Japan) into the rectum, which resulted in laparoscopic low anterior resection without ileostomy.

## Case presentation

**Figure 1:**
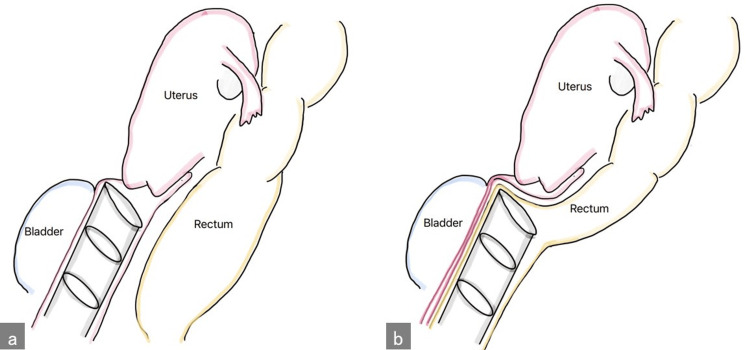
Insertion of a Vagi-Pipe (a) Correct insertion of a Vagi-Pipe. (b) Incorrect insertion of a Vagi-Pipe. Original image created by the authors using Paint (Microsoft Corp., Redmond, WA, US).

A 55-year-old female patient presented with cervical intraepithelial neoplasia. After conization, the pathological diagnosis was severe dysplasia, and the endocervical margin was positive. Therefore, TLH was considered as an additional treatment option. The intraoperative findings were as follows. The uterus was a chicken egg shape, and the bilateral ovaries and tubes were normal. A manipulator was not used because her vagina was narrow owing to nulliparity. After the bladder was dissected from the vaginal wall, the Douglas pouch was dissected from the rectum, and the spatula was inserted into the vagina (Figure 1a). After determining the incision line of the vaginal wall, the second assistant surgeon inserted a Vagi-Pipe, which is 35 mm in diameter, into what appeared to be the vagina (Figure 1b).

**Figure 2:**
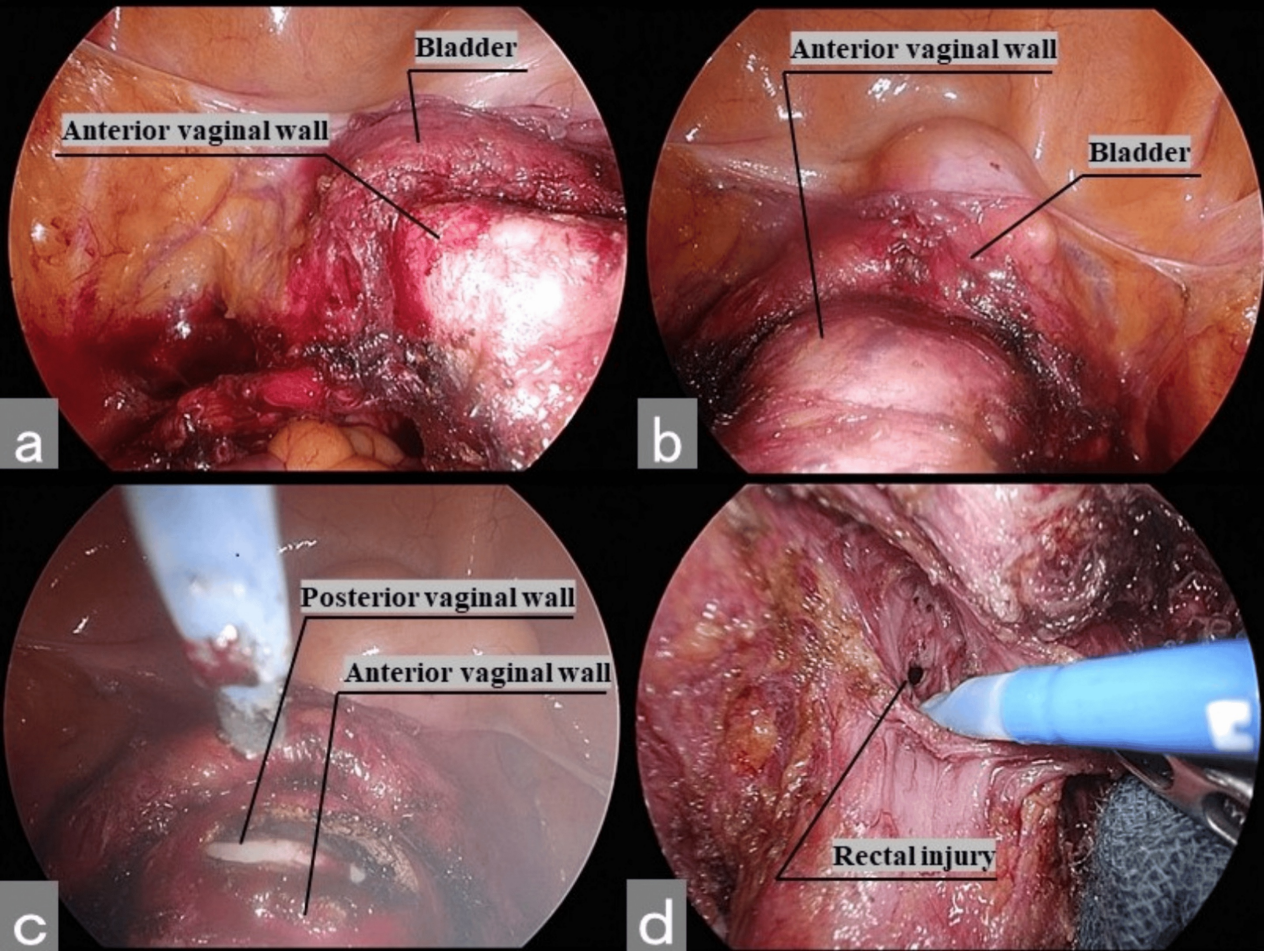
Intraoperative findings (a) Correct insertion of the spatula in the vagina to check the incision line. (b) Incorrect insertion of the Vagi-Pipe to the rectum. (c) Vaginal posterior wall incision. (d) Rectal injury.

Because both the surgeon and first assistant surgeon paid attention only to the monitor screen, they incorrectly confirmed that the Vagi-Pipe was inserted into the vagina (Figure 2a). The Vagi-Pipe was inserted into the rectum because the second assistant misidentified the vaginal orifice and anus (Figure 2b). Owing to the incorrect insertion of the Vagi-Pipe, when the vaginal wall was incised as usual, the rectal mucosa was damaged following injury to the posterior vaginal wall (Figures 2c, 2d).

After consultation with a colorectal surgeon, laparoscopic low anterior resection of the rectum was performed without ileostomy. Postoperatively, she was able to eat meals and had no bowel dysfunction without anastomotic leakage. The postoperative course was uneventful. One year after this surgery, there were no problems with her health.

## Discussion

This is a case of rectal injury during TLH caused by the incorrect insertion of a vaginal pipe. To avoid colostomy, we performed laparoscopic low anterior resection rather than simple suturing.

It has been reported that TLH is associated with complications involving pelvic organs, such as the ureter and rectum [5]. Rectal injury is a rare but potentially serious complication of laparoscopy, and most rectal injuries are related to rectal invasion or severe adhesions due to deep endometriosis [1,11]. However, there have only been a few reports on rectal perforation due to incorrect instrument insertion [12].

There are two main causes of this complication. First, we did not realize that the vaginal pipe was inserted into the rectum before the initial incision. Then, we usually use the Vagi-Pipe to cut the vagina. At our institution, a spatula was inserted into the vagina before inserting the Vagi-Pipe to confirm that the vaginal canal was sufficiently exposed and to determine the incision line of the vaginal canal. In this case, the second assistant surgeon correctly inserted the spatula into the vagina under direct supervision. However, the Vagi-Pipe was incorrectly inserted into the rectum during the next step. The surgeon and first assistant surgeon paid attention to the monitor during this procedure, and the second assistant surgeon used the Vagi-Pipe to push up the posterior vaginal wall, creating the same surgical field as when the Vagi-Pipe is inserted correctly. Once the Vagi-Pipe enters the rectum, it is easy to induce rectal injury because the vaginal canal incision continues until there is direct contact between the electric scalpel and the Vagi-Pipe. Second, we did not consider the effects of muscle relaxation in the anal canal under general anesthesia. In addition, in this case, the vaginal orifice was narrow because of the patient being nullipara. If there is any resistance to the insertion of the Vagi-Pipe into the narrow vaginal orifice, the tip of the Vagi-Pipe slips and is relatively easily inserted into the loose anus just below the vaginal orifice. Based on this experience, at our institution, the anus is covered with a cloth to prevent the assistant from misidentifying the vaginal orifice and anus.

Rectal injury rates have been reported to range between 0.16% and 1.5%. The risk factor of rectal injury in gynecologic laparoscopic surgery is adhesions caused by deep endometriosis and other reasons. The management of rectal injury during TLH has not yet been established. Intraoperative management strategies for accidental rectal injuries include primary repair and low anterior resection. The type of injury (i.e., sharp, blunt, and cautery) is important. In addition, the location of the rectal injury is considered important in surgical management [10]. The advantage of primary repair is that it can be performed in a short period of time, and if no postoperative complications occur, there is less burden on the patient. However, the perforation of the anterior rectal wall is contralateral to the mesenteric vessels, and primary repair may cause intestinal ischemia. A two-layer closure is required to treat rectal injury, and rectal injury of this case is hard to confirm the mucosa. Moreover, the extent of thermal injury caused by the use of an electric scalpel cannot be determined intraoperatively [11]. The difficulty of a two-layer closure is one of the factors in choosing laparoscopic low anterior resection. There are no reports about the treatment for rectal injury between laparoscopic low anterior resection and simple suturing. In contrast, general surgeons in our institution are familiar with low anterior resection, as it is often performed for rectal cancer, which minimizes the risks of ischemia and thermal injury. Considering suture failure occurs in 10% of cases and the risks of ischemia and thermal injury, we selected low anterior resection without colostomy in this case.

## Conclusions

We encountered a case in which laparoscopic low anterior resection was performed for a rectal injury caused by the incorrect insertion of a Vagi-Pipe into the rectum. However, the management of rectal injury during TLH has not yet been established, and we have to choose the management according to the type of rectal injury. If the spatula had been correctly inserted into the vagina, the Vagi-Pipe may have been inserted into the rectum by the second assistant due to various factors. Surgeons performing TLH should always consider the possibility of incorrect insertion of the Vagi-Pipe, which can lead to rectal injury.
